# The Argument Web: an Online Ecosystem of Tools, Systems and Services for Argumentation

**DOI:** 10.1007/s13347-017-0260-8

**Published:** 2017-05-11

**Authors:** Chris Reed, Katarzyna Budzynska, Rory Duthie, Mathilde Janier, Barbara Konat, John Lawrence, Alison Pease, Mark Snaith

**Affiliations:** 1 Centre for Argument Technology, University of Dundee, Dundee, DD1 4HN UK; 2 Centre for Argument Technology, IFiS PAN, ul. Nowy Swiat 72, 00-330 Warszawa, Poland

**Keywords:** Argumentation, Argument mining, Debate technology, Dialogue games, Mixed initiative argumentation

## Abstract

The Argument Web is maturing as both a platform built upon a synthesis of many contemporary theories of argumentation in philosophy and also as an ecosystem in which various applications and application components are contributed by different research groups around the world. It already hosts the largest publicly accessible corpora of argumentation and has the largest number of interoperable and cross compatible tools for the analysis, navigation and evaluation of arguments across a broad range of domains, languages and activity types. Such interoperability is key in allowing innovative combinations of tool and data reuse that can further catalyse the development of the field of computational argumentation. The aim of this paper is to summarise the key foundations, the recent advances and the goals of the Argument Web, with a particular focus on demonstrating the relevance to, and roots in, philosophical argumentation theory.

## Introduction

The growth in computational models of argument over the past two decades has been phenomenal and represents a success story in humanities-sciences interdisciplinary research. Fragmentation in the area, however, has consistently threatened to undermine its maturity with reduced incremental development and a litany of wheel re-inventions. This is the problem that has been tackled by the Argument Web: to provide an environment or ‘ecosystem’ in which data sharing and re-use, incremental development of software, and theory and application interoperability are the quotidian modus operandi of research in the field. This paper aims to do two things: first, to provide an overview of the Argument Web and its various foundations, applications, tools and datasets for an audience that is more familiar with philosophical ground; and second, to show how theories of argumentation developed in philosophy, communication, linguistics and social psychology have influenced and been taken up in both the core engineering that has brought the Argument Web into being, and also in its various applications and software systems that are starting to drive usage.

The approach in the paper is necessarily broad and shallow. We begin in Section 2 with central concepts of argument representation, first for common argument concepts, then extensions particularly for dialogue (this separation reflects discussions in the philosophical literature stretching back to the early 1980s on the relationship between argument products and argument processes). The extended Section 2 thus represents the underpinning upon which the remainder of the paper depends. The first port of call for the application of these argument representations is in the philosophically very familiar task of argument analysis, in Section 3. The introduction of analytical techniques, however, is divided by the rather engineering-oriented distinction between individual and collaborative analysis. For philosophy, and particularly critical thinking and informal logic, the most prominent use of argument analysis techniques is in pedagogy and pedagogical application of the Argument Web; these are reviewed in Section 4. Driven in part by the social epistemological movement, many areas of philosophy are increasingly being influenced by a empiricist perspective and argumentation is no exception. Section 5 looks at the ways in which the Argument Web can support such research through tools for curation and management of linguistic resources. With so much data available, navigation and evaluation of argument becomes increasingly challenging, and these areas are briefly reviewed in Sections 6 and 7, respectively. An extraordinarily hot topic in computational models of argument is currently the challenge of argument mining—automatically extracting argument structures from natural text; some of the approaches and successes are reviewed briefly in Section 8 and then finally in Section 9 an example from the philosophy of science (and specifically of mathematics) is summarised to show how many of these pieces can be brought together to achieve significant theoretical advances. Across this breadth, the paper aims to equip the reader with both theoretical and practical understanding of the Argument Web.

## Argument Representation

The interdisciplinary overlap between philosophical and computational approaches to argumentation has been a major growth area within Artificial Intelligence over the past three decades. AI has long been an idiosyncratic hybrid of pure theory and pragmatic engineering, and nowhere is this more true than in computational models of argument. The mathematical theories of argument which originate in works such as that by Dung (1995) have been enormously influential in theoretical models of reasoning in AI, because they provide the machinery for handling issues such as defeasibility and inconsistency in ways that traditional classical logics are not able to support. These same mathematical theories are, however, barely recognisable as theories of argumentation as the philosophical and communication scholarly communities would know them—they serve rather as ‘calculi of opposition’.^1^

At the same time, AI is also home to applications of theories of informal logic (Gordon et al. 2007), of pedagogic critical thinking (Reed and Rowe 2004), of rhetoric (Crosswhite et al. 2003) and of legal argumentation (Walton 2005): these applications are all rooted squarely in the tradition of argumentation theory as a discipline, and diverge from it in ways that are typically incremental and driven by pragmatic necessity.

Whilst the fecundity of the research area has been clear (Rahwan and Simari 2009), the diversity and sheer number of different systems has led, inevitably, to fragmentation. It was this problem that led in 2005 to a workshop to explore possible means of harmonisation between approaches and systems. The remit of the meeting was avowedly practical: to try to find ways that these systems might start to work together. But practical, engineering issues turn very quickly to deep and open philosophical issues: What constitutes an enthymeme, a fallacy or an inference? What differentiates presumptions and assumptions in argument? How can linguistic and psychological conceptions of argument be reconciled? Are propositions the right atoms from which to construct argumentation complexes? What is the character of the rules that govern argument dialogues? And so on.

Clearly, it is impractical to hope that these questions might be resolved once and for all, so the approach is to start by focusing, quite pragmatically, upon what is currently the best understanding of the various issues. Walton’s work figures large here (Walton 1984, 1996; Walton and Krabbe 1995). But the work has also tapped into pragma-dialectics, into speech act theory, and into the work of theorists such as Brockriede (1974), Freeman (1991), Groarke (1999), Hitchcock (2002), Kienpointner (1992), Krabbe (2003), O’Keefe (1977) and Perelman and Olbrechts-Tyteca (1969)^2^ amongst many others.

At the first iteration, the goal is to lay some solid foundations with a focus specifically upon representing arguments. Whilst there are many AI systems that reason with arguments, present arguments, render arguments in natural language, try to understand natural arguments, visualize arguments, navigate arguments, critique arguments, support the construction of arguments, mediate arguments, and so on, we cannot hope to begin by solving problems special to each. It seems reasonable to assume, however, that all of these systems might want to store arguments in some structured format. This, then, is the focus. If we want to set out to try to support harmonisation between systems, and to do so in a way that is as closely tied as possible to current models from the theory of argumentation, then we start with a simple task that is common across most AI systems of argument: representation.

### The Argument Interchange Format

The Argument Interchange Format (AIF) (Chesñevar et al. 2006) was developed to provide a flexible—yet semantically rich—way of representing argumentation structures. The AIF was put together to try to harmonise the strong formal tradition initiated to a large degree by Dung (1995), the natural language research described at CMNA workshops since 2001,^3^ and the multi-agent argumentation work that has emerged from the philosophy of Walton and Krabbe (1995), amongst others.

The AIF can be seen as a representation scheme constructed in three layers. At the most abstract layer, the AIF provides a hierarchy of concepts which can be used to describe argument structure. This hierarchy describes an argument by conceiving of it as a network of connected nodes that are of two types: information nodes that capture data (such as datum and claim nodes in a Toulmin analysis, or premises and conclusions in a box-and-arrow analysis in the style of Freeman (1991), for example), and scheme nodes that describe passage between information nodes (similar to the application of warrants or rules of inference). Scheme nodes in turn come in several different guises, including scheme nodes that correspond to support or inference (or ‘rule application nodes’), scheme nodes that correspond to conflict or refutation (or ‘conflict application nodes’) and scheme nodes that correspond to value judgements or preference orderings (or ‘preference application nodes’). At this topmost layer, there are various constraints on how components interact: information nodes, for example, can only be connected to other information nodes via scheme nodes of one sort or another. Scheme nodes, on the other hand, can be connected to other scheme nodes directly (in cases, for example, of arguments that have inferential components as conclusions, e.g. in patterns such as Kienpointner’s (1992) ‘warrant-establishing arguments’). Inference captured by multiple incoming scheme nodes thus naturally corresponds to convergent argumentation; that covered by multiple premises supporting a single incoming scheme node corresponds to linked argumentation (Walton 2006). The AIF also provides, in the extensions developed for the Argument Web (Rahwan et al. 2007), the concept of a ‘Form’ (as distinct from the ‘Content’ of information and scheme nodes). Forms allow the AIF to represent uninstantiated definitions of schemes (this has practical advantages in allowing different schemes to be represented explicitly—such as the very rich taxonomies of Walton et al. (2009), Perelman and Olbrechts-Tyteca (1969), Grennan (1997), etc.—and is also important in law, where arguing about inference patterns can become important).

A second, intermediate layer provides a set of specific argumentation schemes (and value hierarchies, and conflict patterns). Thus, the uppermost layer in the AIF ontology lays out that presumptive argumentation schemes are types of rule application nodes, but it is the intermediate layer that cashes those presumptive argumentation schemes out into Argument from Consequences, Argument from Cause to Effect and so on. At this layer, the form of specific argumentation schemes is defined: each will have a conclusion description (such as ‘*A* may plausibly be taken to be true’) and one or more premise descriptions (such as ‘*E* is an expert in domain *D*’). Walton’s schemes (Walton 1996; Walton et al. 2009) have been developed in full for the AIF (Rahwan et al. 2007).

It is also at this layer that, as Rahwan et al. (2007) have shown, the AIF supports a sophisticated representation of schemes and their critical questions. In addition to descriptions of premises and conclusions, each presumptive inference scheme also specifies descriptions of its presumptions and exceptions. Presumptions are represented explicitly as information nodes, but, as some schemes have premise descriptions that entail certain presumptions, the scheme definitions also support entailment relations between premises and presumptions. The AIF has here largely followed the lead of a collaboration between Walton and two AI researchers, Gordon and Prakken (Gordon et al. 2007).

Finally, the third and most concrete level supports the integration of actual fragments of argument, with individual argument components (such as strings of text) instantiating elements of the layer above. At this third layer, an instance of a given scheme is represented as a rule application node (with the terminology of rule application—RA—and conflict scheme application—CA—and so on now easier to interpret). This rule application node is said to fulfill one of the presumptive argumentation scheme descriptors at the level above. As a result of this fulfillment relation, premises of the rule application node fulfill the premise descriptors, the conclusion fulfils the conclusion descriptor, presumptions can fulfill presumption descriptors, and conflicts can be instantiated via instances of conflict schemes that fulfill the conflict scheme descriptors at the level above. Again, all the constraints at the intermediate layer are inherited, and new constraints are introduced by virtue of the structure of the argument at hand.

The AIF standard is available in a number of different ‘reifications’—that is, in a number of different computer languages, from detailed and extensive data expressed in a language compatible with the Semantic Web (viz., RDF and OWL), through versions in languages familiar to programmers in commercial and scholarly domains such as JSON and Prolog, as well as compact languages and formats aimed, for example, at visualisation (such as DOT and SVG).

In addition, various threads of research have demonstrated equivalences (often with some limiting or simplifying assumptions) with major other computational approaches to argumentation such as the high profile Carneades tool which has a jurisprudential foundation and offers rich evaluative mechanisms (Gordon et al. 2007); the popular analysis tool Rationale (van Gelder 2007); one of the most mature approaches to formal representation of ‘structured’ (in contrast to ‘abstract’ argumentation) in ASPIC + (Bex et al. 2013); and a specific form of abstract argumentation that grounds arguments in observations, Evidence-based Argumentation Frameworks or EAFs (Oren et al. 2010).

### Extensions to Handle Dialogue

The next step is to allow the representation of dialogue. Several preliminary steps in this direction have been taken (Reed 2006; Modgil and McGinnis 2008; Reed et al. 2008), building on work in computational systems on protocol specification (see, e.g. the work of the FIPA group^4^) and in philosophy on dialogical games (such as Mackenzie 1990). The motivation for this work can be summarised through O’Keefe’s distinction between *argument*_1_ and *argument*_2_: *argument*_1_ is an arrangement of claims into a monological structure, whilst *argument*_2_ is a dialogue between participants—as O’Keefe (1977)[p122] puts it, ‘The distinction here is evidenced in everyday talk by ... the difference between the sentences ‘I was *arguing*_1_ that P’ and ‘we were *arguing*_2_ about Q.’ ’ There are self-evident links between *argument*_1_ and *argument*_2_ in that the steps and moves in the latter are constrained by the dynamic, distributed and inter-connected availability of the former, and further in that valid or acceptable instances of the former can come about through sets of the latter. An understanding of these intricate links which result from protocols and argument-based knowledge demands a representation that handles both *argument*_1_ and *argument*_2_ coherently. It is this that the dialogic extensions to the AIF set out to provide.

The fundamental building blocks of dialogues are the individual locutions. In the context of the AIF, Modgil and McGinnis (2008) have proposed modelling locutions as information nodes. We follow this approach primarily because statements about locution events are propositions that could be used in arguments. So for example, the proposition, *Bob says, ‘ISSA will be in Amsterdam’* could be referring to something that happened in a dialogue (and later we shall see how we might therefore wish to reason about the proposition, *ISSA will be in Amsterdam*) – but it might also play a role in another, monologic argument (say, an argument from expert opinion, or just an argument about Bob’s communicative abilities).

Associating locutions exactly with information nodes, however, is insufficient. There are several features that are unique to locutions, and that do not make sense for propositional information in general. Foremost amongst these features is that locutions often have propositional content. The relationship between a locution and the proposition it employs is, as Searle (1969) argues, constant – i.e. “propositional content” is a property of (some) locutions. Whilst there are other, non-locution propositions that may also relate to further propositions, (e.g. the proposition, *It might be the case that it will rain*) the relationship of propositional content is certainly not ubiquitous (*It is Tuesday* does not have propositional content—it simply *is* a proposition). On these grounds, we should allow representation of locutions to have propositional content, but not demand it for information nodes in general – and therefore the representation of locutions should form a subclass of information nodes in general. We call this subclass “locution nodes”. There are further reasons for distinguishing locution nodes as a special case of information nodes, such as the identification of which dialogue(s) a locution is part of. (There are also some features which one might expect to be unique to locutions, but on reflection are features of information nodes in general. Consider, for example, a time index—we may wish to note that Bob said, *ISSA will be in Amsterdam* at 10am exactly on the 1st March 2010. Such specification, however, is common to all propositions. Strictly speaking, *It might be the case that it will rain* is only a proposition if we spell out where and when it holds. In other words, a time index could be a property of information nodes in general, though it might be rarely used for information nodes and often used in locution nodes).

Given that locutions are (a subclass of) information nodes, they can, like other information nodes, only be connected through scheme nodes. There is a direct analogy between the way in which two information nodes are inferentially related when linked by a rule application, and the way in which two locution nodes are related when one responds to another by the rules of a dialogue. Imagine, for example, a dialogue in which Bob says, *ISSA will be in Amsterdam* and Simon responds by asking, *Why is that so?*. In trying to understand what has happened, one could ask, ‘Why did Simon ask his question?’ Now although there may be many motivational or intentional aspects to an answer to this question, there is at least one answer we could give purely as a result of the dialogue protocol, namely, ‘Because Bob had made a statement’. That is to say, there is plausibly an inferential relationship between the proposition, *Bob says ISSA will be in Amsterdam* and the proposition, *Simon asks why it is that ISSA will be in Amsterdam*. That inferential relationship is similar to a conventional inferential relationship, as captured by a rule application. Clearly, though, the grounds of such inference lie not in a scheme definition, but in the protocol definition. Specifically, the inference between two locutions is governed by a *transition*, so a given inference is a specific application of a transition. Hence, we call such nodes “transition application nodes” and define them as a subclass of rule application nodes. (Transition applications bear strong resemblance to applications of schemes of reasoning based on causal relations: this resemblance is yet to be further explored, but further emphasises the connection between inference and transition).

So, in just the same way that a rule application fulfils a rule of inference scheme form, and the premises of that rule application fulfil the premise descriptions of the scheme form, so too, a transition application fulfils a transitional inference scheme form, and the locutions connected by that transition application fulfil the locution descriptions of the scheme form. The result is that all of the machinery for connecting the normative, prescriptive definitions in schemes with the actual, descriptive material of a monologic argument is re-used to connect the normative, prescriptive definitions of protocols with the actual, descriptive material of a dialogic argument.

With these introductions, the upper level of this extended AIF is almost complete. For both information (I-) nodes and rule application (RA-) nodes, we need to distinguish between the old AIF class and the new subclass which contains all the old I-nodes and RA-nodes excluding locution (L-) nodes and transition application (TA-) nodes (respectively). As the various strands and implementations of AIF continue, we will want to continue talking about I-nodes and RA-nodes and in almost all cases, it is the superclass that will be intended. We therefore keep the original names for the superclasses (I-node and RA-node), and introduce the new subclasses I^′^ and RA^′^ for the sets I-nodes ∖L-nodes and RA-nodes ∖TA-nodes respectively.

One final interesting question is how, exactly, L-nodes are connected to I-nodes. So for example, what is the relationship between a proposition *p* and the proposition ‘X asserted *p*’? According to the original specification of the AIF, direct I-node to I-node links are prohibited (and with good reason: to do so would introduce the necessity for edge typing—obviating this requirement is one of the advantages of the AIF approach). The answer to this question is already available in the work of Searle (1969) and later with Vanderveken (Searle and Vanderveken 1985). The link between a locution (or, more precisely, a proposition that reports a locution) and the proposition (or propositions) to which the locution refers is one of illocution. The illocutionary force of an utterance can be of a number of types (Searle and Vanderveken (1985) explore this typology and its logical basis in some detail) and can involve various presumptions and exceptions of its own. In this way, it bears more than a passing resemblance to scheme structure. These schemes are not capturing the passage of a specific inferential force, but rather the passage of a specific illocutionary force. As a result, we refer to these schemes as *illocutionary schemes* or Y schemes. Specific applications of these schemes are then, following the now familiar pattern, illocutionary applications or YA nodes. Illocutionary schemes are referred to with gerunds (asserting, promising, etc.), whilst transitional inference schemes are referred to with nouns (response, statement, etc.), which both ensures clarity in nomenclature, and is also true to the original spirit and many of the examples in both the Speech Act and Dialogue Theory literatures.

### Further Extensions

As a common interlingua for representing argumentation, the AIF thus captures a simple core of argument-related notions. Whether working in philosophy, linguistics or computer science, it is inevitable that specific research projects, teams or schools will need to go beyond this lowest common denominator. For that reason, the AIF infrastructure has been designed to support not only a core vocabulary (or “ontology”), but also a principled mechanism by which it can be extended with additional, supplementary representation systems (or “adjunct ontologies”, AOs). AOs are designed according to a general guiding principle that they should encapsulate the AIF core. One of the most mature AOs available for the Argument Web is the social layer, a set of components for maintaining information about arguers and users of argumentation software (Snaith et al. 2013). Applications that make use of the social layer (such as Argublogging (Snaith et al. 2012)) access the social layer only; the social layer encapsulates all of the information available in the AIF. In this way, AIF resources can continue to be shared between all Argument Web systems, whilst the specific needs of individual sets of applications with specific requirements can be catered for appropriately. (Of course, in many cases, including that of the social layer AO, there are also multiple applications which can share these extended data sets – OVA and Arvina, discussed later, both make use of the social layer, for example).

### Examples of the AIF in Action

**Fig. 1:**
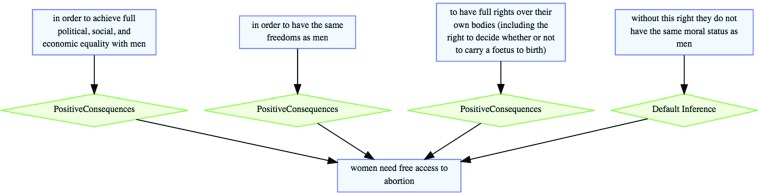
Three instances of positive consequences reasoning (from aifdb.org/argview/2307)

To show how the AIF handles familiar types of argumentative structures and exchanges, we include here three examples: a Walton argumentation scheme; a Pollock-style undercutter and a dialogical exchange. In Fig. 1, the claim *women need free access to abortion* forms the conclusion of four convergent arguments. Three correspond to instances of the argumentation scheme for Argument from Positive Consequences, plus a further argument not assigned any specific scheme in this analysis. For each instance, it is possible to reconstruct the implicit components associated with the scheme, such as, e.g. *achieving full political, social and economic equality with men is desirable*. In this way, the implicit premises of an argument can be identified by matching up explicitly mentioned structure with characteristic structure in the general scheme definition. The template provided by the scheme points the way to the implicit premises. Such reconstruction has been demonstrated to be a particularly tricky task (Hitchcock 2002), so this template-driven enthymeme resolution is theoretically valuable. Schemes also embody *critical questions* which, as explored by Verheij and Arguments (2005) and then extended in the AIF by Rahwan et al. (2007), play several roles, indicating not just implicit components, but also stereotypical patterns of attack. Such patterns of attack are of several kinds and one of the most important distinctions is that between rebutting and undercutting attack. Whilst conflict between claims can straightforwardly capture rebutting attack, the structure of undercutting attack is a little more complex. The notion of undercutting was introduced by Pollock (1995) so we use his example to explain the AIF approach to undercutting.

**Fig. 2:**
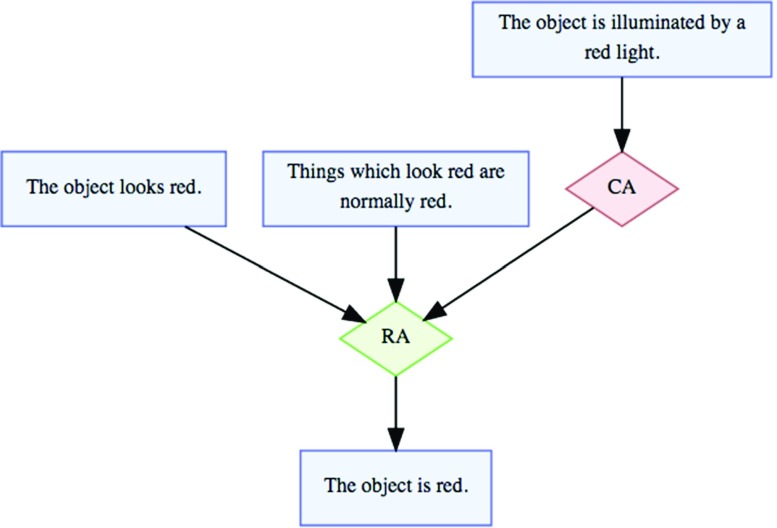
Undercutting in the AIF (from aifdb.org/argview/4)

In Fig. 2, the conclusion that the object appears red is not itself attacked, but rather the inference (from the premise that it appears red to the claim that it is red) is attacked. Thus, the conflict (the Conflict Application, or CA) targets the inference (the Rule of inference Application, or RA). As both Verheij and Arguments (2005) and Rahwan et al. (2007) explore, some critical questions associated with schemes are associated with rebutting structures, some with undercutting structures.

Finally, the AIF has also been extended to handle argument that is situated in dialogical situations (Reed et al. 2008). A key insight in that work takes issue with, for example, the pragma-dialectical claim that illocutionary acts of arguing are located at the point of premise-giving (van Eemeren and Grootendorst 1992), which relies upon interpreting premise-giving as a complex speech act. By reifying the rules that govern dialogical progression, and then permitting those rules themselves to create or ‘anchor’ illocutionary force, arguing can be interpreted as an illocutionary act that comes about as the result of a relation between uttering a premise and uttering a conclusion, thus mirroring the logical structure of inference in its illocutionary structure. This theoretical approach that underpins the dialogical extensions to AIF is known as Inference Anchoring Theory (Budzynska and Reed 2011).

**Fig. 3:**
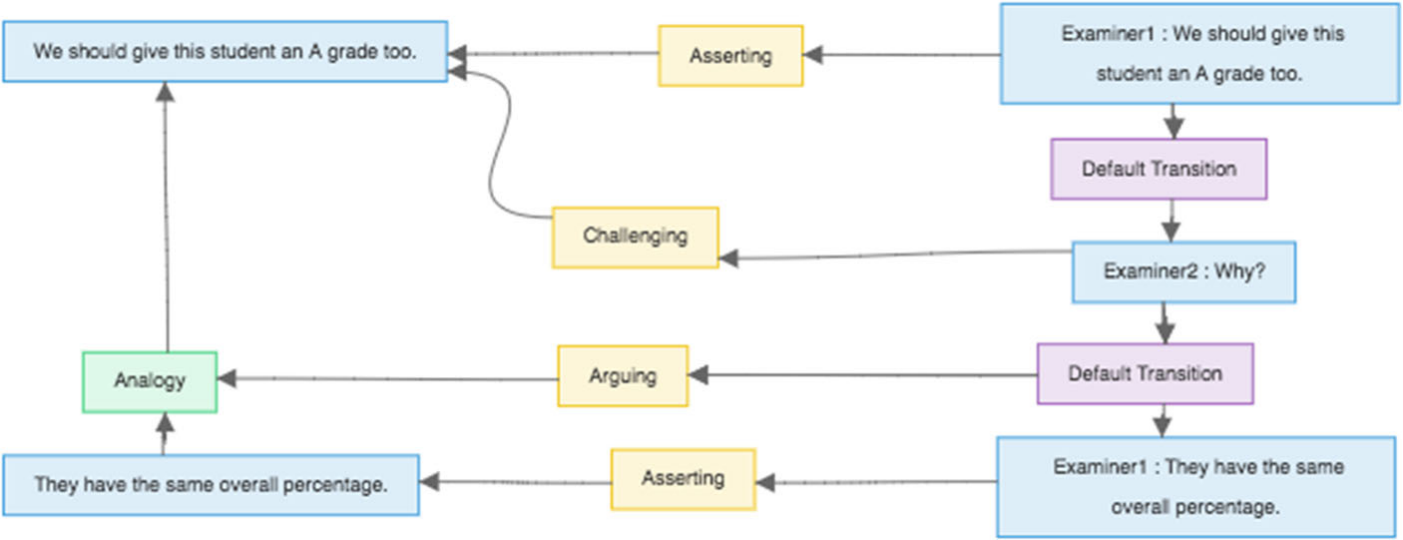
Dialogue in the AIF (from the OVA+ view accesible from aifdb.org/argview/10939)

Figure 3 shows how this works: dialogical activity is shown on the right hand side, connected by applications of rules of dialogue (Transitions, or TAs) such as that a challenge can be responded to by a substantiating assertion. Simple locutions can anchor illocutionary forces—*Why?*, for example, anchors *challenging* (the propositional content of which is the same as the preceding assertion). But TAs can also anchor illocutionary force: the connection between the challenge and its response is the locus of arguing (in this case, the illocutionary force of arguing has as its content an instance of the argumentation scheme for Argument from Analogy).

## Argument Analysis

### Individual Analysis

With techniques for representing argument, perhaps the first place to start is how to exploit that representation language and create argument data—this is the task of argument analysis. There is a long history of argument analysis software which has in turn spawned a number of review and comparison articles including the work of Harrell (2005), Scheuer et al. (2010) and Kirschner et al. (2003) which review different application areas (including deliberation, eDemocracy and eRulemaking, the law, and so on) and different philosophical starting points (Toulmin, Freeman, Walton and more). The emergence of the AIF was itself a result of this tradition, with the markup language used by Araucaria (Reed and Rowe 2004) used as a base, enhanced with features from Carneades (Gordon and Walton 2006), DeLP (García and Simari 2014) and others, reflecting not ony practical improvements but also improvements reflecting deeper theoretical insights (including the ability to handle undercutting arguments, full graph structures, etc.).

Between 2001 and 2010, Araucaria was a widely used analysis tool, with over 10,000 users in 80 countries. Though implementing a rather primitive conception of argument, it was the first tool to cater for multiple analytical styles or theories of argument—not only a common ‘box and arrow’ analysis characterised in the most detail by Freeman (1991), but also a view based on Toulmin’s account of argument (Toulmin 1958) and in addition a further view embodying a rich diagrammatic vocabulary developed by the legal theorist Wigmore (1931). Crucially, despite the differences in the visual conventions, the underlying data structures and the XML-based language for storing them was unified with theory-specific extensions where theoretical elements were not mappable to concepts in other approaches. This established the concept of ‘theory neutrality’ (Reed and Rowe 2005) which remains a cornerstone of the Argument Web. That is, there are sufficient features of argumentation common to most or all theories and conceptions to form a common interlingua.

A number of systems built upon the success of Araucaria, including language specific developments such as an open-source branch of the code base in Mandarin, and a programme of published research in Polish (Budzynska 2011).

More significantly, however, most of the functionality of Araucaria was re-implemented for online, in-browser use in the OVA (Online Visualisation of Argument) tool.^5^ Though diagramming in Toulmin and Wigmore styles is not available (for these, Araucaria is the only option), OVA supports a range of analytical goals.

### Collaborative Analysis

Most software tools designed to support argument analysis focus on a single user. Software such as Google Docs, however, has demonstrated the strong appetite for tools that support collaborative working; with OVA running online, it was extended in version 2.0 to provide a Google-style link to allow multiple analysts to work together on a single analysis (Janier et al. 2014). A number of other online tools (such as DebateGraph, debategraph.org and RationaleOnline, rationaleonline.com) support multiple concurrent users, but few support collaborative argument analysis.

**Fig. 4:**
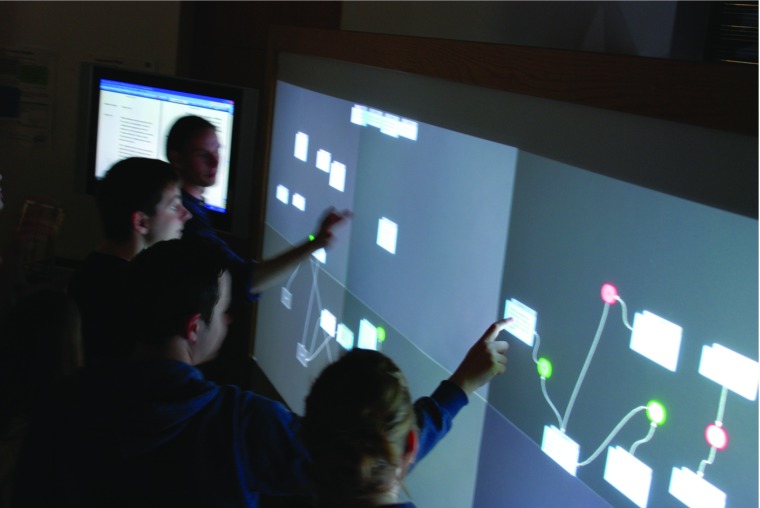
The AnalysisWall

Some types of analysis demand large-scale collaborative analysis. For working with live broadcast debate, the AnalysisWall (Fig. 4) provides a large, shared workspace—a very high resolution 7.7 sqm touchscreen running bespoke argument analysis software supporting effectively unlimited concurrent touch points (i.e. with no restrictions other than physical space on the number of analysts that can work to gether).^6^ The analysis that is supported is similar to that offered by OVA: connecting nodes through inferences and conflicts, optionally specifying argumentation schemes, distinguishing undercutting from rebutting and marking reported speech. In addition, the very size of the AnalysisWall (at over 3m long) means that it is important to support grouping of nodes so that an analyst standing to the lefthand side can share some work by flicking an analysed group across to an analyst working on the right. The AnalysisWall supports collaborative analysis streamlined to work in real-time and has been used to conduct analysis of the BBC Radio 4 programme *The Moral Maze*. Coupled with a stenographer who could provide a live text stream, plus two analysts working on a separate terminal to segment the text into argumentative components which were then delivered automatically to the Wall, the hardware enabled teams of between six and ten analysts to work together, just managing to keep pace with the incoming flow of argumentation from the radio programme. The team would split and internally restructure briefly and fluidly, forming ad hoc groups of two or three to work on a part of the reasoning before returning to the overall analysis. This analysis resulted in large AIF datasets all of which are then manipulable by the other tools desribed in this paper. Results from this analysis can be found at, for example, aifdb.org/argview/789.

## Argument Pedagogy

One of the largest domains and strongest drivers for work on software for argument analysis has been pedagogy (though pedagogical applications often go beyond just analysis). In many areas of the world, the teaching of critical thinking forms a key part of a broad introductory foundation to university level education, and in almost all parts of the world, definitions of university ‘graduateness’ refer to skills normally considered a part of critical thinking or informal logic.

The various tools for analysis described in the previous Section have all been deployed in educational settings. In particular, OVA is widely used currently with around 5000 unique users per year from over 50 countries.

One of the attractions of deploying Argument Web tools in the classroom is that there are also software applications for providing automatic assessment and feedback. Previously, the only way of making use of automated mechanisms for grading and assessment was to use multiple-choice examinations (Fisher and Scriven 1997). Such tests are not ideal and cannot be used across the board, however, because they assess only a narrow range of skills, offering only very limited scope for assessing students’ depth of understanding. Many software systems have focused instead on allowing students to conduct detailed box-and-arrow style analyses (for a review and detailed comparison see, e.g., Scheuer et al. 2010)—the problem is that such analyses have had to be marked by hand.

**Fig. 5:**
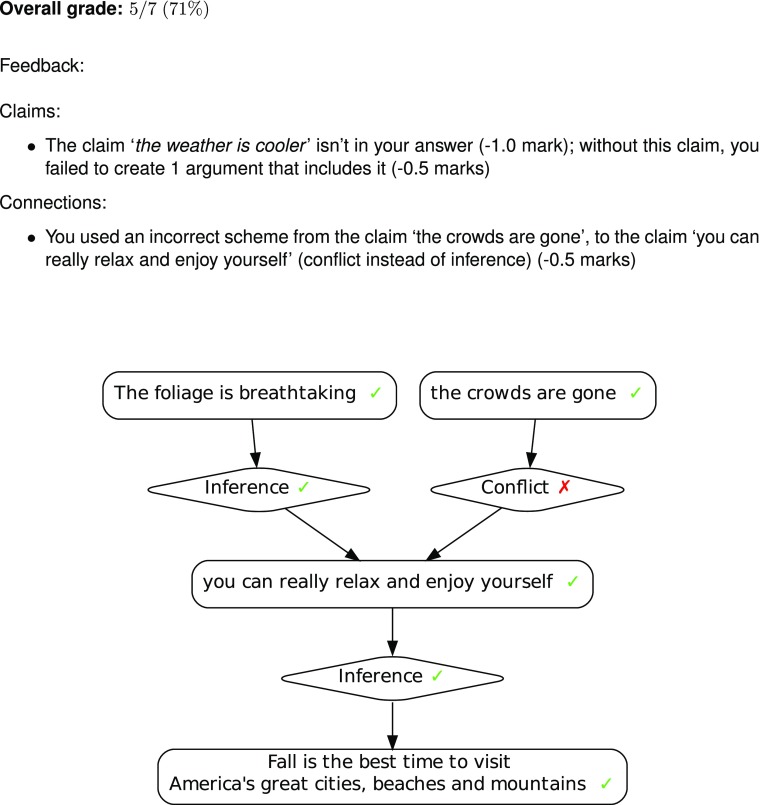
Feedback from the Argugrader system

Argument Web infrastucture, however, allows such student analyses to be easily manipulated by other software components. By running graph matching algorithms over student analyses prepared in OVA, it is possible to compare submissions against one or more model answers, converting results not only into a grade (via a tutor-configured scoring algorithm) but also template-driven textual feedback. This is the functionality offered by the Argugrader system^7^ which has been trialled both at the University of Dundee and at City University of New York. An example of the feedback offered by Argugrader is shown in Fig. 5 showing component-by-component marking of a student generated analysis, plus template-driven feedback and overall scoring. To the best of our knowledge, this is the only extant system that automatically performs grading on the basis of argument structure.

## Argument Curation

Alongside an increasing engagement with empiricism in philosophy, the use of data has started to play a more significant role in argumentation theory (see, for example, Goodwin’s panel on empirical and corpus research in argumentation at the International Pragmatics Conference in 2007). As a result, the Argument Web has a variety of features to facilitate the collection, curation, sharing, tracking, comparison and summary of datasets.

AraucariaDB (Reed 2005) was the first publicly available corpus of analysed argumentation in the world, but was rather limited in size (around 700 arguments) and analysis rubric (with no measurement of inter-annotator agreement). Though AraucariaDB was imported into the Argument Web infrastructure, it now forms just one corpus amongst many.

At the beginning of 2017, the servers hosted by the Centre for Argument Technology for providing aifdb.org 8 stored 11,000 arguments involving 60,000 claims expressed in around 1.2m words. The infrastructure supports many different scripts (and has been tested with Mandarin, Korean, Hindi, Arabic, Hebrew and Cyrillic), and the databases store analyses of arguments that have been contributed in 14 different languages.^9^

As the size of the dataset increases, organisation is required. Sharing of argument analyses is crucial to support the growth of the community because such analysis is so labour intensive. However, research teams need to have confidence in the permanent availability of resources, of their immutability, that they can be cited, aggregated and reused. This is the challenge of data curation for the Argument Web, and it is to tackle this challenge that AIFdb was extended to support definition of *corpora*. These extensions are available online at corpora.aifdb.org. Many of the data available in AIFdb are now organised into specific corpora, where individual corpora have been developed for particular research projects or in particular research groups. Again at the start of 2017, there are over 70 corpora developed by nine different labs in Europe and North America, covering fields of argumentation as diverse as mediation, pedagogy, politics, broadcast debate, eDemocracy and financial discussion. In addition, two other corpora both of which became available in 2014: *viz.* the Potsdam Microtext Corpus (Peldszus and Stede 2015) and the Internet Argument Corpus (Walker et al. 2012) have been migrated to the Argument Web infrastructure. In order to meet the needs of the community, corpora can be freely defined to package up sets of analyses (or indeed sets of subcorpora) from AIFdb. Corpora may be locked by their creators so that they are immutable—though other users could create similar corpora with newer contents of course. At time of writing, the tools for managing Argument Web corpora are undergoing a process which will allow corpora to be allocated Digital Object Identifiers (DOIs) allowing them to be formally citeable in their own right.

**Fig. 6:**
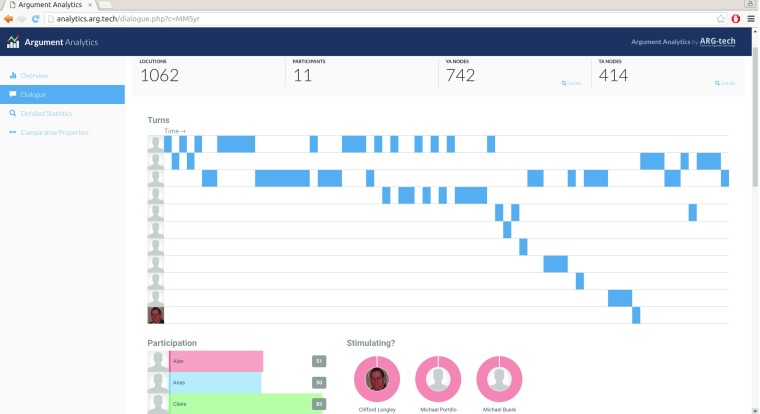
Analytics for the Argument Web

As a common research task, the statistical analysis of corpora is supported through a suite of analytics (at analytics.arg.tech, Fig. 6) that offer insight into the size and structure of corpora, and allow comparison between them (for measurements such as the *κ* estimate for inter-annotator agreement (Fleiss 1971)).

## Argument Navigation

With many thousands of argument resources available on the Argument Web infrastructure, and the rate of addition continuing to increase, there is an increasingly acute need for tools to help navigate and interrogate the data. There are three broad types of navigation: static, dynamic and dialogical.

Static navigation of argument resources comprises straightforward visualisation techniques of graph structures and ‘point-and-click’ navigation traversing from one claim in an argument network to another. Though good quality interface design makes this a convenient mechanism for dealing with small argument networks (of up to around 100 component parts—premises, conclusions, etc.), it rapidly becomes unmanageable and confusing as the datasets become greater. An average analysis of a 45-minute episode of the BBC Radio 4 debate programme, *The Moral Maze*, for example, comprises around 500 components; viewing a programme in its entirety renders text illegible and interconnections little more than spaghetti (see, for example, aifdb.org/argview/789).

For those arguments naturally associated with a temporal flow, an obvious means of navigation is using the time axis to scroll back and forward through the discourse. Inspired by the graphically extraordinary work of Pluss and De Liddo (2015), tools for temporal playback are now being released for dialogical AIF resources.

Finally, an observation offered by Freeman (1991) has laid a foundation for a further type of navigation. Freeman suggests that the relationships between components in a (perhaps entirely monological) argument can be probed by considering what questions might have been asked to yield the relationships. Thus, a convergent argument structure is associated with a fictitious respondent asking, ‘Can you give me another reason?’ whilst a linked structure is associated with the question, ‘Why is that relevant?’ Though Freeman’s goal was to shed light upon the tricky structural distinction, his method can be operationalised in software as a navigation tool. That is, we can treat dialogue as a means of navigating the information space defined by argument structures. Unfortunately, we cannot assume that there is a single set of dialogue moves or dialogue rules by which we might navigate any argument. Different contexts, different domains, different fields may demand different types of dialogue. So for example if we are navigating legal arguments, perhaps navigation is best achieved in part through questions concerning the burden of proof; for arguments being navigated in pedagogical settings, perhaps questions that elicit partial answers or that require more specificity for the student are most appropriate. Such dialogical navigation can thus be translated by software into varying sets of instantiated moves from which users can select at any point and sets of less constrainted moves that allow new information to be elicited from users.

As a result, the Argument Web provides a language for describing the rules of these dialogue games or dialogue protocols (essentially, this is a specialised computer programming language)—the Dialogue Game Description Language, DGDL (Wells and Reed 2012). Many well known philosophical investigations from Ramus to the present day have made use of formal dialectical games; Wells and Reed (2012) show how many of these games (Hamblin’s H, Mackenzie’s DC, Walton’s CB, Walton & Krabbe’s PPD and others) can be captured as DGDL specifications.

With many different dialectical games available in a library of DGDL specifications, a general purpose dialogue execution engine is required to run them. This is provided by the Dialogue Game Execution Platform, DGEP, which provides a simple environment that software engineers can use to build applications that support human-human or human-machine dialogue. There are several such applications available, the two most stable of which are Arvina and Argublogging.

**Fig. 7:**
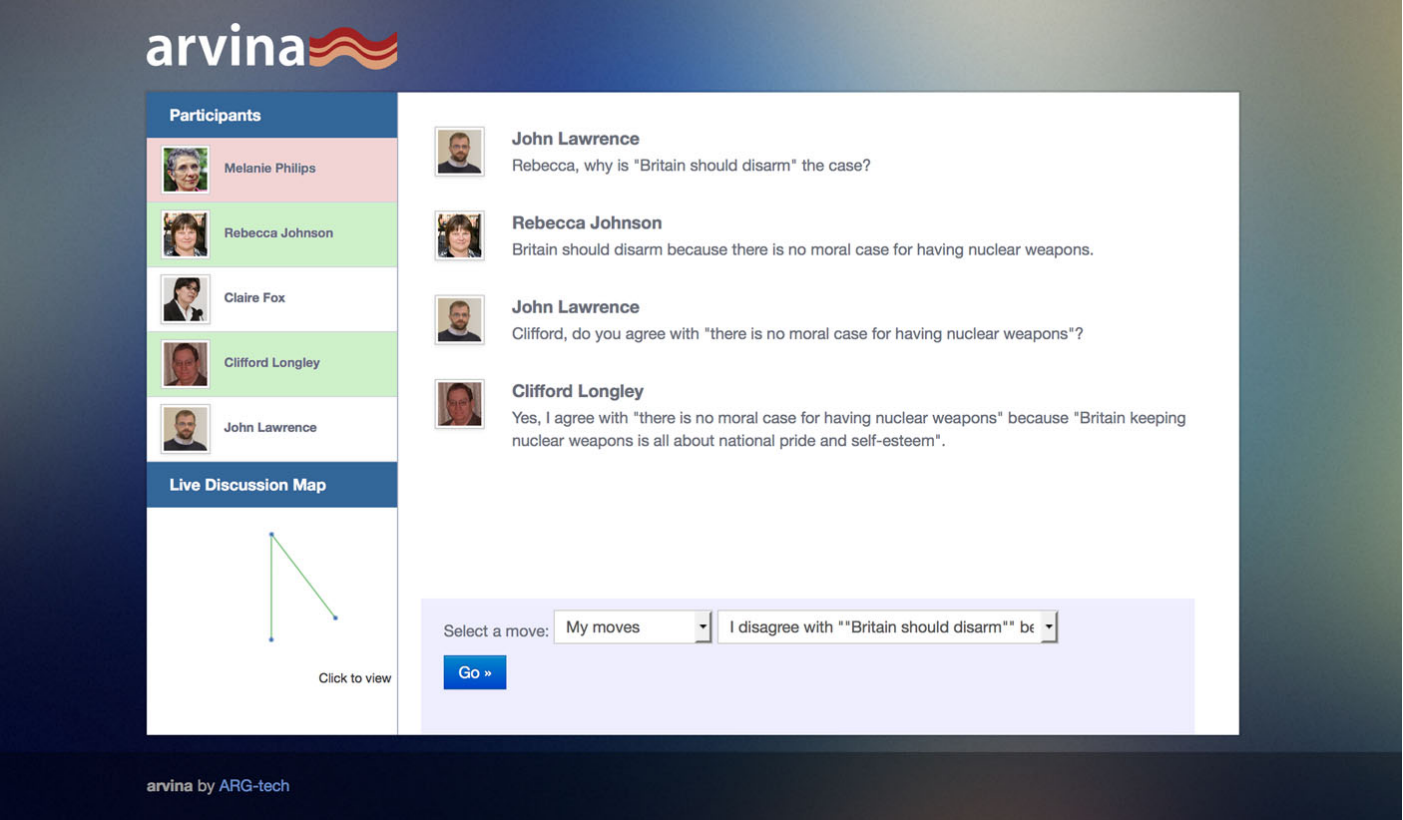
Dialogue on the Argument Web with Arvina

Arvina allows multiple humans to engage in a structured dialogue simultaneously with mutiple software agents. (Arvina and DGEP make no distintion between human players and artificial players; they simply keep a track of who can say what when—this ‘level playing field’ between humans and software in dialogue is known as *mixed initiative argumentation*). The software agents are responsible for arguments already available on the Argument Web and associated with a specific individual who originally put them forward. In this way, software agents can advocate on behalf of the original proponents. Conversely, human participants can use the dialogue game to explore the arguments of previous, human participants (or arguments that have been analysed using tools such as OVA). Figure 7 shows Arvina in use.

Another dialogue application on the Argument Web is designed specifically for bloggers, enabling arguments to be co-constructed between bloggers and between blogs. The system is described in more detail in the work of Snaith et al. (2012), but what is salient here is the way in which dialogue games do not just prescribe how information is navigable, but also, at the same time, how it is to be extended. To extend Freeman’s questions, above, if a dialogue game allows a user to express their opinion and for some other agent to ask for a reason and why that reason is relevant, then as an inevitable side effect, the Argument Web is updated not just with the user’s opinion, but with the opinion structured as a linked argument. Argublogging embodies an extremely simple dialogue game, but nevertheless provides an interface that is intuitive for bloggers whilst at the same time intercepts their contributions so that the structured arguments on their blogs are available through AIF on the Argument Web. Such user-generated content holds great potential as a way of gathering large amounts of structured data for further scholarly research.

## Argument Evaluation

Both computational and philosophical exploration of argument place great emphasis on the ability to evaluate arguments. In philosophy, such evaluation is founded upon standards of proof, or levels of audience acceptability, or persuasiveness or rationality, or by reference to a set of normative standards. In computational work, evaluation has been largely focused on analytical definitions of acceptability. This notion is akin to consistency, although, via the work of Dung (1995) on abstract argumentation acceptability, acceptability semantics is significantly more robust in the face of sub-deductive reasoning (such as inductive, presumptive and defeasible patterns) common in natural argumentation.

The Argument Web offers a variety of evaluation engines. Because of the strong focus in AI on evaluation over argument structures, this is an area of the Argument Web that is leading the way in terms of functioning as an ecosystem in which different research labs contribute different parts of solvers that combine to become available in general purpose form. Dung-O-Matic (Snaith et al. 2010) was an early implementation of a variety of acceptability semantics (including grounded, preferred, ideal, eager, and semi-stable) that works only on abstract frameworks. TOAST (Snaith and Reed 2012) works with structured data (i.e. raw AIF structures), converting them to abstract frameworks according to Bex et al. (2013) and then calling Dung-O-Matic. Tweety (Thimm 2014) is an implementation of DeLP (García and Simari 2014) that uses a simple mapping from AIF into a logic program. Finally ArgSemSAT (Cerutti et al. 2014) uses a computational technique known as SAT solving to compute acceptability over abstract frameworks using the same translation as is performed in TOAST.

## Argument Mining

If the Argument Web is to expand its reach and increase its data resources by several orders of magnitude, techniques for manual analysis and for intercepting user generated content will not be enough. It will be necessary to turn to automated techniques for identifying both the presence and the structure of argument in unrestricted natural text. This is the challenge of argument mining.

Just a few years ago, the prospect of automatically extracting the structure of reasoning from natural language text was firmly beyond the state of the art: only very preliminary work was being carried out at a small number of research groups such as Leuven, Dundee, and Toronto (Feng and Hirst 2011; Moens et al. 2007). Now, there are at least twenty research groups across the US and the EU, in which work on the problem has begun in earnest, with three workshops, including one at the major computational linguistics conference, ACL, during the summer of 2014. The rapid expansion is due in equal measure to the increasing maturity of computational techniques (such as those for argument annotation using supervised learning and topic-based argument structure recovery using unsupervised learning) and clear commercial demand in areas such as financial market prediction and marketing research.

Though Palau and Moens (2011) offer a good introduction, the field is moving so quickly that the best reviews of the area are currently offered by the tables of contents of the 2014, 2015 and 2016 ACL workshops on Argument Mining (see argmining2016.arg.tech).

## Case Study

Pease et al. (2017) show how AI techniques for understanding abstract argumentation and its connections to structured argumentation, linguistic expression of reasoning and dialogical practice can work together in a complete cycle, from real-world argument to philosophical account, formal theory, abstract argumentation and argumentation semantics, and back to application to real-world argument. Extending earlier work (Pease et al. 2014), we describe each stage in the cycle both formally and our implementation of it, employing a range of argument tools described above.

**Fig. 8:**
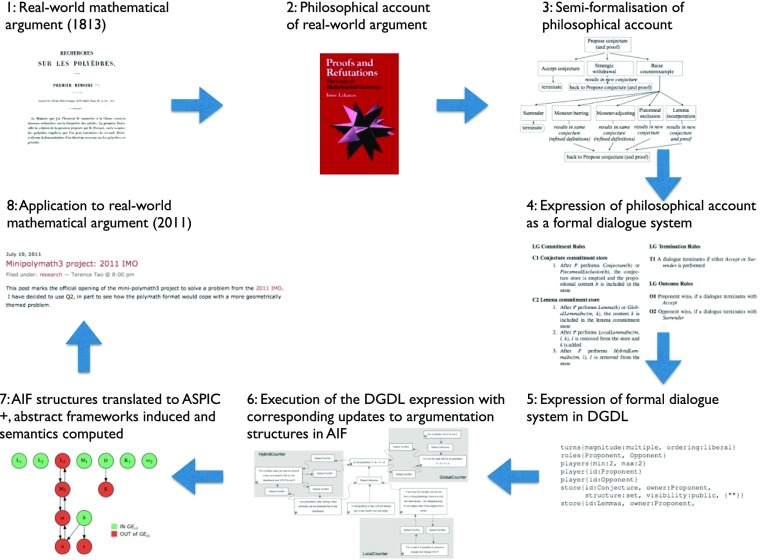
The stages involved in the cycle from real-world argument to Lakatos’s account of mathematical argument, to our model and implementation and application to further real-world argument

Firstly, we take an existing philosophical account of ways in which mathematical theories evolve via interactions between mathematicians who might have differing motivations, background theories, concept definitions and so on—specifically, Lakatos’s account (Lakatos 1976), which is based on real world historical case studies (1 and 2 in Fig. 8). We then express this account as a formal dialogue system with sets of locution, structural, commitment and termination rules (3, 4, 8), and express this in a domain specific language for dialogue game specifications (5). This can be executed using DGEP and we define operational semantics in terms of updates to argumentation structures expressed in a linguistically oriented ontology for argumentation, AIF (6). Using TOAST, DungOMatic, and AIFdb, abstract argumentation frameworks are then automatically induced from AIF via the structured argumentation system ASPIC+ (Modgil and Prakken 2014), showing the consequences of AIF updates at the abstract layer and demonstrating how those abstract semantics yield a grounded extension that provably always corresponds to the theory that has been collaboratively created by the dialogue participants (7)—that is to say, the rules of the dialogical game defined by Lakatos correspond precisely to the rules of argumentation-based reasoning defined by Dung and others. Bringing the stages full-circle, we show how the model accounts for real mathematical dialogues online (8). Closing the circle in this way, starting and ending with real-world mathematical discourse, allows us not only to demonstrate the depth of Lakatos’s original insight, but also to show that the formal characterisation here remains both honest to the original and of practical utility to mathematicians. By making this connection back to the community of argumentation practice, the door is opened to mixed-initiative, collaborative mathematics.

This is the first time that formally specified and fully implemented argumentation tools right through the abstraction hierarchy from linguistic expressions through structured argumentation to instantiated abstract argumentation have been brought together and applied to a specific, demanding domain of human reasoning. The foundation that has been laid here allows new explorations into mixed-initiative, collaborative reasoning between human and software participants in interactions which are both naturalistic but formally constrained and well-defined, with the potential to impact both the pedagogy and the professional practice of mathematics.

## Concluding Remarks

The key challenge facing the Argument Web now is one of evaluation. Evaluation of these component parts of the Argument Web cannot simply be expressed as a series of psychological experiments or usability studies. Each component has various facets each of which can be explored separately. The evaluation of argument representation, for example, can be conducted with respect to theoretical adequacy (does it handle specific philosophical concepts in a way that remains consistent with theoretical results and predictions?); inter-annotator agreement (do analysts agree on how to analyse specific examples when trained to a particular skill level?); computational expressivity (does it allow for representation of sufficiently common complex examples?). Similarly, computation of values given argument structures might proceed with respect to intuition (that is to say, do automatic computational processes deliver the same results as humans, and are artificially computed results consistent with psychological experimentation?); mathematical models (do computational processes deliver the same results as those modelled mathematically in systems such as Dung’s abstract argumentation?); epistemological definitions (do computational processes deliver results that are plausible on epistemological grounds, or that fit epistemological theory?). And so on. This evaluative programme represents a major undertaking for the community and one that will spur on the development of both the Argument Web in particular and the field of computational argumentation in general.

As things stand, the Argument Web comprises a broad set of tools, datasets, infrastructure, working policies, interfaces, standards and research programmes that is not only facilitating the coherence and maturity of research in computational models of argument, and the availability of data for training AI algorithms in argument mining, but also serving as a proving ground and perhaps also an inspirational playground for the development and testing of philosophical theories of argumentation. The Argument Web is thus not only a product of the fruitful interaction between AI and the philosophy of argument to date, but also stands to facilitate the growth of that interdisciplinary connection in the future.

## Footnotes

1 The phrase ‘calculus of opposition’ is due to Henry Prakken: he suggested it in a private discussion, but it strikes us as a particularly apt description not only of what is supported by Dung’s concept of acceptability, but also very much the spirit behind it.

2 These citations are indicative: there are both many more works by these authors and many other authors that have had an impact on computational developments.

3
http://www.cmna.info

4 Available online at http://www.fipa.org/repository/aclspecs.html.

5 OVA is available at ova.arg-tech.org

6 The hardware for this was developed on a shoestring, with six budget projectors providing imaging onto a hand-built Frustrated Total Internal Reflection touch surface with feedback detected by modified Playstation cameras. More information about the build is available at arg.tech/analysiswall

7 See www.argugrader.com

8 AIF databases can be hosted locally and federated.

9 Chinese, Dutch, English, French, German, Hindi, Italian, Japanese, Korean, Polish, Portuguese, Russian, Spanish and Ukrainian
